# Generation of Full-Length cDNAs for Eight Putative GPCnR from the Cattle Tick, *R. microplus* Using a Targeted Degenerate PCR and Sequencing Strategy

**DOI:** 10.1371/journal.pone.0032480

**Published:** 2012-03-05

**Authors:** Sean W. Corley, Emily K. Piper, Nicholas N. Jonsson

**Affiliations:** 1 School of Veterinary Science, The University of Queensland, Gatton, Queensland, Australia; 2 College of Medical, Veterinary and Life Sciences, The University of Glasgow, Glasgow, United Kingdom; University of Kentucky College of Medicine, United States of America

## Abstract

We describe here a rapid and efficient method for the targeted isolation of specific members of gene families without the need for cloning. Using this strategy we isolated full length cDNAs for eight putative G-protein coupled neurotransmitter receptors (GPCnR) from the cattle tick *Rhipicephalus* (*Boophilus) microplus*. Gene specific degenerate primers were designed using aligned amino acid sequences of similar receptor types from several insect and arachnid species. These primers were used to amplify and sequence a section of the target gene. Rapid amplification of cDNA ends (RACE) PCR was used to generate full length cDNA sequences. Phylogenetic analysis placed 7 of these sequences into Class A G-protein coupled receptors (GPCR) (Rm_α2AOR, Rm_β2AOR, Rm_Dop1R, Rm_Dop2R, Rm_INDR, Rm_5-HT_7_R and Rm_mAchR), and one into Class C GPCR (Rm_GABA_B_R). Of the 7 Class A sequences, only Rm_mAchR is not a member of the biogenic amine receptor family. The isolation of these putative receptor sequences provides an opportunity to gain an understanding of acaricide resistance mechanisms such as amitraz resistance and might suggest possibilities for the development of new acaricides.

## Introduction

The isolation of specific members of gene families typically involves the use of degenerate primers designed in regions conserved across many members of the gene family of interest. The subsequent PCR product requires cloning, as it contains amplicons from multiple genes. Clones are then sequenced looking for the gene of interest. This strategy is labour intensive, time consuming and often fails to isolate the target gene. We describe here a rapid, targeted approach which enables isolation of the gene of interest without cloning, using a degenerate PCR and sequencing strategy based on homologous amino acid motifs specific to each target gene.

GPCR are characterised by 7 trans-membrane spanning domains (TM) that contain the ligand-binding site, an extracellular amino-terminus, and an intracellular carboxyl-tail [1]. Ligand binding to the GPCR causes interactions with G proteins, mediating a series of intracellular functional responses via the second messengers, adenyl cyclase and or phospholipase C [2]. Between one third [3] and a half [4] of currently marketed human drugs, target GPCR. The cattle tick, *Rhipicephalus (Boophilus) microplus* is a very important parasite of cattle throughout the world and its control relies heavily on acaricides. The pyrethroid, formamidine and macrocyclic lactone families of acaricides are among the most widely used acaricides for controlling cattle ticks at present; all of which are known or believed to target membrane-associated proteins, potentially including GPCR. To date only two G-protein coupled neurotransmitter receptors have been described in the cattle tick [5], [6]. We believe that *R.microplus* GPCR involved in the regulation of vital physiological functions offer valuable targets for new acaricides. Here we report the targeted isolation of eight putative G-protein coupled neurotransmitter receptors from the cattle tick, *R. microplus*.

## Results

**Figure 1:**
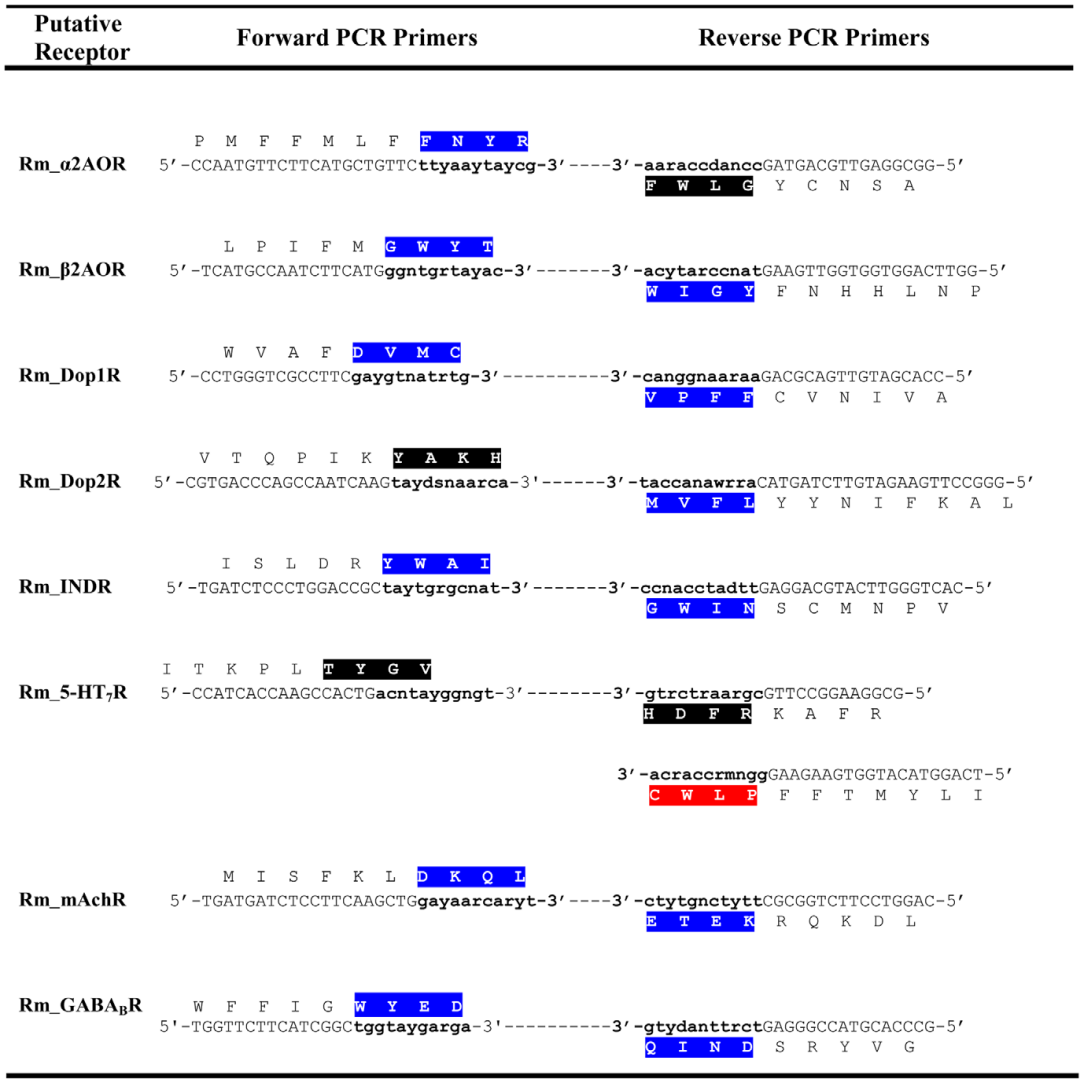
Degenerate PCR and sequencing primers designed using CODEHOP software. Nucleotides comprising the 3′ degenerate tail are in small letters. Nucleotides forming the 5′ non-degenerate clamp are in large letters. Amino acids comprising the degenerate tail are highlighted. Primers with the degenerate tail highlighted in black were used for PCR only. Primers with the degenerate tail highlighted in blue were used for both PCR and sequencing. The primer with the degenerate tail highlighted in red was an internal degenerate sequencing primer required for clean sequence.

**Figure 2:**
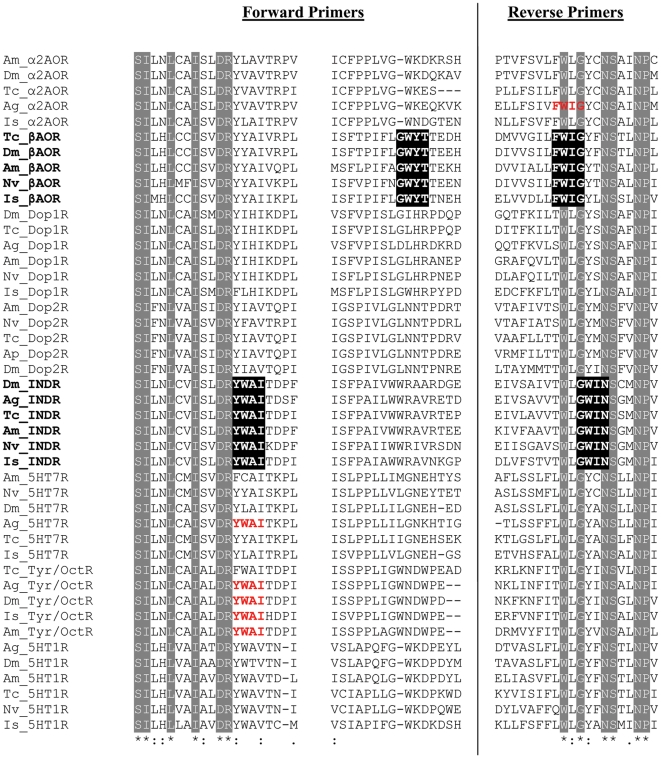
Selected sections of sequences from an alignment of biogenic amine receptors. Conserved motifs used to design specific degenerate primers for INDR and βAOR receptors are highlighted in black. The occurrence of these conserved motifs in non target receptor types are in red. Amino acids conserved across all receptors are highlighted in grey. Ag-*Anopheles gambiae*, Am-*Apis mellifera*, Ap-*Acyrthosiphon pisum*, Dm-*Drosophila melanogaster*, Is-*Ixodes scapularis*, Nv-*Nasonia vitripennis*, Tc-*Tribolium castaneum.* Sequences accession numbers are listed in Table S1.

**Figure 3:**
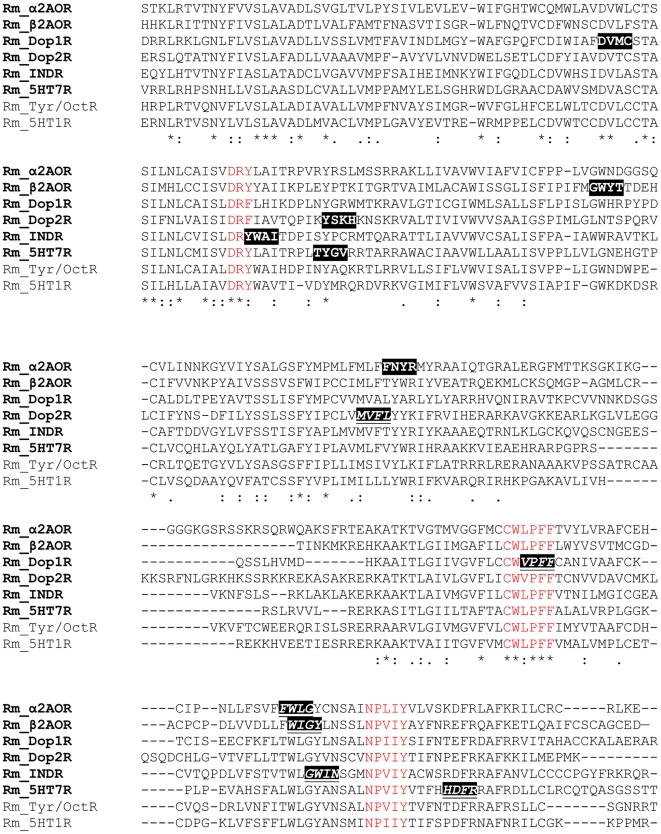
Positions of forward and reverse degenerate primers for the six *R. microplus* biogenic amine receptors. The four amino acids comprising the degenerate tail of the primers are highlighted illustrating the gene specific nature of the primer design. Reverse primers are italicised and underlined. Examples of motifs highly conserved across receptor types, used for isolation by the alternative method of degenerate PCR and cloning, are in red. Sequences isolated in this manuscript are in bold.

**Figure 4:**
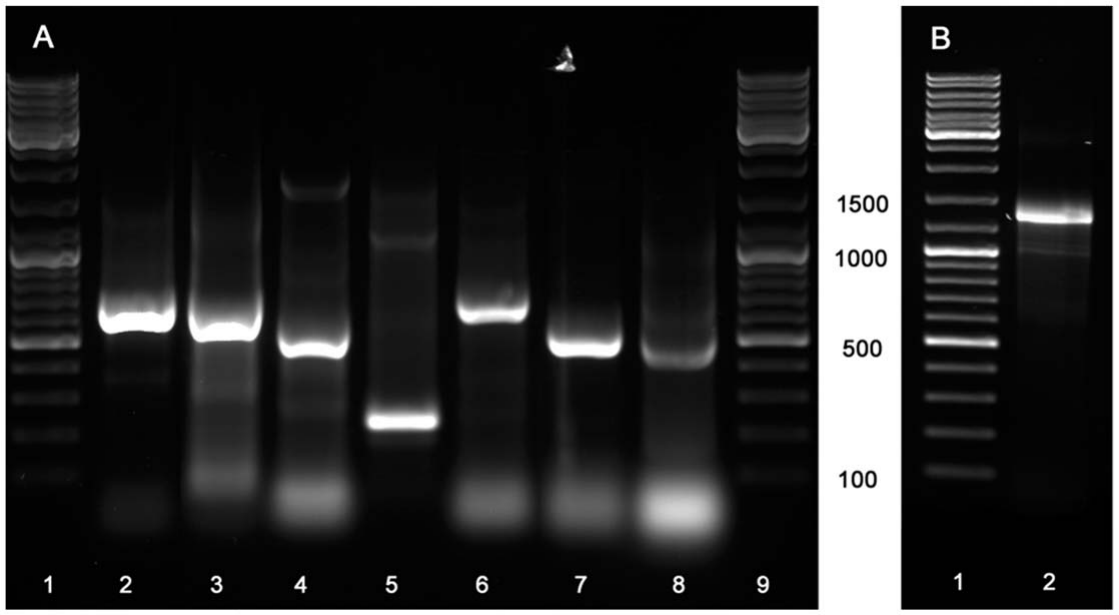
Degenerate PCR products from genomic DNA for the 7 putative Class A and Class C GPCR. **A**) Lanes, 1 and 9_DNA ladder, 2_ Rm_α2AOR, 3_ Rm_Dop1R, 4_ Rm_β2AOR, 5_Rm_Dop2R, 6_Rm_INDR , 7_Rm_5HT_7_R, 8_Rm_mAchR. **B**) Lanes, 1 _DNA ladder, 2_ GABA_B_R.

**Figure 5:**
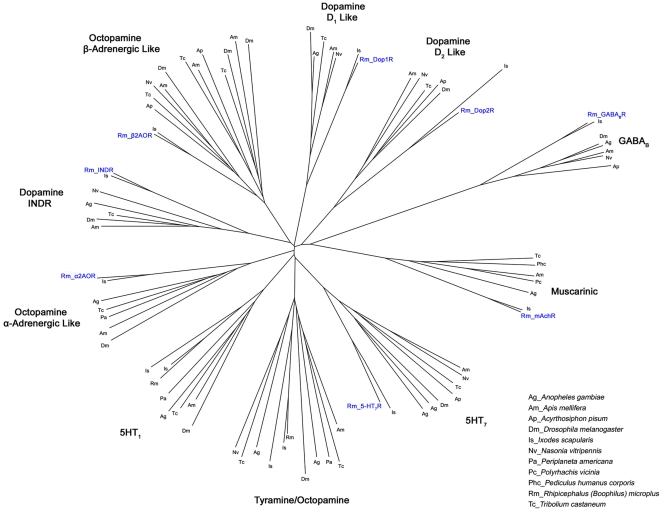
A phylogenetic tree of insect and arachnid G-protein coupled neurotransmitter receptor sequences. Amino acid sequences were aligned using Clustalw2 (European Bioinformatics Institute, Cambridge, UK). Sequence accession numbers are listed in Table S2. The radial phylogram was constructed using Dendroscope software [33]. Dopamine receptor nomenclature is as proposed by Mustard *et al.*, (2005). Octopamine receptor nomenclature is as proposed by Evans *et al.*, (2005). Previously undescribed putative *R. microplus* receptors are in larger blue type.

Using the gene specific degenerate primers designed with CODEHOP software (Figures 1, 2, 3), fragments from the eight target genes were amplified from both genomic (gDNA) and cDNA. The amplicons ranged in size from 234–1277 bp (Figure 4) and were the same size for both gDNA and cDNA indicating that each degenerate primer pair was contained within one exon. Sequencing with degenerate primers yielded a single clean sequence from each amplicon. Following RACE PCR eight complete coding sequences were generated. Through phylogenetic analysis, these sequences could be grouped into two distinct classes of GPCR; Class A GPCR (or Rhodopsin like GPCR) and Class C GPCR. Figure 5 shows a phylogenetic diagram of insect and arachnid GPCR, including the 8 putative receptors reported in this paper. These receptors have been submitted to GenBank under the following accession numbers. Class A) Rm_α2AOR (JN974908), Rm_β2AOR (JN974909), Rm_Dop1R (JN974914), Rm_Dop2R (JN974912), Rm_INDR (JN974911), Rm_5-HT_7_R (JN974910) and Rm_mAchR (JN974913). Class C) Rm_GABA_B_R (JN974907).

### Class A GPCR

Seven of the 8 sequences were grouped as Class A GPCR. These were the putative octopamine, dopamine, 5-hydroxytryptamine (5-HT_7_) and muscarinic acetylcholine (mAch) receptors. As well as the 7 trans-membrane (TM) spanning domains (Figure S1) typical of all GPCR, Class A receptors share a number of conserved features. There is an Asp–Arg–Tyr (DRY) or Asp–Arg–Phe (DRF) motif located in the second intracellular loop. This motif is important for the conformational changes involved in receptor activation. It is highly conserved among the Class A GPCR; sometimes this motif is also present as Glu-Arg-His (ERH) [7] or as Glu-Arg-Tyr (ERY) [8] but in all cases, within this class, the Arg residue is conserved. This motif is believed to be located in a hydrophilic pocket formed by polar residues from TM I, TM II and TM VII. Agonist binding causes protonation of the aspartic or glutamic acid residue, causing the Arg residue to move out of the pocket [9]. Two cysteines, one present in the first extracellular loop and the second in the second extracellular loop, are expected to form a disulphide bond which stabilizes the receptor [10], [11]. All these features are present in the 7 sequences grouped in Class A (Figures S2 & S3).

#### Biogenic Amine GPCR

Six of the 7 Class A receptors belong to the biogenic amine receptor family. These are the putative octopamine, dopamine and 5-HT_7_ receptors. In addition to features common to all Class A GPCR, characteristics typical of biogenic amine receptors include the presence of an aspartate (D) in trans-membrane domain TM III. This acts as a counter-ion for binding of the amine group. Two serines in TM V form hydrogen bonds with the catechol hydroxyl groups, and a phenylalanine in TM VI interacts with the catechol aromatic ring [12]. An aspartate residue in TM II which is involved in receptor activation is also conserved (Figures S3 & S4). These features are present in the 6 putative biogenic amine receptors with the exception of the 5-HT_7_ receptor, in which the two serines in TM V are replaced by two alanines. The other 5-HT receptor (5-HT_1_ like) previously isolated from *R. microplus* [6] contain a serine and alanine at these positions (Figure S4).

#### Putative Octopamine Receptors

Two of the newly generated sequences were phylogenetically most similar to octopamine receptors, which have been proposed as the target of amitraz [13], [14].This class of receptor is preferentially activated by octopamine. Octopamine is a major neurotransmitter, neuromodulator and neurohormone, regulating diverse physiological processes in invertebrates, including fight or flight responses, egg-laying, sensory information processing, and complex neural functions such as learning and memory [15]. Sequence JN974908 (Rm_α2AOR ) is most similar to α-adrenergic-like octopamine receptors (αAOR). These receptors show the greatest similarity structurally and pharmacologically to vertebrate α-adrenergic receptors. They mediate their effects via increases in intracellular calcium levels [16]. Sequence JN974909 (Rm_β2AOR) is most similar to β-adrenergic-like octopamine receptors (βAOR). This class of receptors shows the greatest similarity structurally and pharmacologically to vertebrate β-adrenergic receptors [17]. In the presence of octopamine, increases in intracellular cAMP levels occur.

#### Putative Dopamine Receptors

Three of the newly generated sequences were phylogenetically most similar to invertebrate dopamine receptors. These receptors are preferentially activated by dopamine. Dopamine has been demonstrated to activate flight motor activity in *Manduca sexta* [18]. Dopamine has also been shown to reduce the response to conditioned stimuli as well as inhibiting retrieval of learned information in *Apis mellifera* [19]. Sequence JN974914 (Rm_Dop1R) is most similar to dopamine type 1 receptors (Dop1R). This class is most closely related to vertebrate D1 receptors and increases intracellular cAMP levels in the presence of dopamine. Sequence JN974912 (Rm_Dop2R) is most similar to dopamine type 2 receptors (Dop2R). These receptors share the closest homology with vertebrate D2 receptors and treatment with dopamine decreases intracellular cAMP. Sequence JN974911 (Rm_INDR) is most similar to invertebrate dopamine receptors (INDR). Although, as with D1 receptors, stimulation with dopamine increases intracellular cAMP levels [20], these receptors are more closely related structurally to invertebrate octopamine receptors.

#### Putative 5-HT_7_ Receptor

Sequence JN974910 (Rm_5-HT_7_R) is most closely related to invertebrate 5-HT_7_ receptors that mediate their effects by increasing intracellular cAMP levels. 5-HT has been demonstrated to enhance circadian rhythm-dependent general motor activity in the moth *Lymantria dispar*, while suppressing dopamine-induced flight motor activity in *Manduca sexta* [18]. It has also been shown to reduce conditioned olfactory responses in the honeybee *Apis mellifera* [19], [21].

#### Putative Muscarinic Acetylcholine Receptor (mAchR)

Sequence JN974913 (Rm_mAchR) is most closely related to invertebrate muscarinic acetylcholine receptors (mAchR). Agonist binding of these receptors typically decreases intracellular cAMP levels by inhibiting adenylate cyclase or stimulating phospholipase C and the turnover of inositol phosphates. Muscarinic agonists have been shown to be effective acaricides [22], [23]. Muscarinic receptors have been credited with two main functions in insects: inhibition of transmitter release from sensory neuron terminals and regulation of the excitability of motoneurons and interneurons [24].

### Class C GPCR

#### Putative Metabotropic γ-Aminobutyric Acid Receptor (GABA_B_R)

A single sequence grouped with Class C GPCR. This was the putative metabotropic GABA_B_ (γ-aminobutyric acid) receptor JN974907 (Rm_GABA_B_R). Features characteristic of Class C GPCR are an N-terminal signal peptide, followed by a region with high sequence similarity to bacterial periplasmic amino acid binding proteins [25]. This constitutes the ligand binding site, located in a large extracellular N-terminal domain. The intracellular carboxy terminus is exceptionally large in all GABA_B_ receptors. This protein segment contains a coiled-coil domain, which was shown to be necessary for the formation of GABA_B_R heterodimers in mammalian receptors [26], [27]. The putative GABA_B_ receptor sequence generated from *R. microplus* contains these features (Figure S5). GABA (γ-aminobutyric acid) functions as the primary inhibitory neurotransmitter in the central nervous system of vertebrates and invertebrates [28]. Stimulation with GABA causes a reduction in intracellular cAMP enabling regulation of K^+^ and Ca^2+^. Ionotropic GABA_A_ receptors are distributed throughout both the central and peripheral insect nervous systems [29] and are important targets for insecticides. Much less work has been done with metabotropic GABA_B_ receptors and they too may offer important insecticide targets.

## Discussion

Next generation sequencing technologies have seen dramatic decreases in cost and time associated with whole genome sequencing. This has seen the increased use of bioinformatics approaches for gene isolation. Despite this, degenerate PCR remains a powerful tool for gene isolation. The technique of designing degenerate primers in regions conserved across many members of the gene family of interest can be likened to a “shotgun” approach where amplicons from many genes are generated in the hope that the gene of interest is among these. Cloning is used to determine if the target gene has been amplified. This “hit or miss” strategy is both labour intensive, time consuming and ultimately may not generate the desired result .We report here on a targeted degenerate PCR and sequencing strategy for gene isolation. This strategy avoids amino acid motifs highly conserved across different gene types when designing degenerate primers and concentrates on motifs conserved only within the same gene type. This technique can be used with both gDNA and cDNA. By using a product such as GenomeWalker™ (Clontech Laboratories Inc. Mountain View, USA) to replace RACE PCR, genes can also be isolated from gDNA enabling gene isolation from samples unsuitable for RNA extraction. Using this process we were able to target and isolate eight putative *Rhipicephalus* (*Boophilus) microplus* G-protein coupled neurotransmitter receptor sequences without the need for cloning.

In other organisms, these GPCR have been shown to be involved in mediating a wide and diverse range of physiological processes. The ability to disrupt or alter these processes forms the basis by which many insecticides and acaricides act. The formamidine acaricide amitraz is believed to target the octopamine receptor, while muscarinic agonists have been shown to be effective acaricides. Ionotropic GABA_A_ receptors are important targets for insecticides while only limited information about metabotropic GABA_B_ receptors is available. 5-HT and dopamine receptors are also believed to mediate important physiological processes and may offer targets for new acaricides. An important consideration when developing new acaricides are their possible toxic effects on non-target organisms. The isolation of these putative receptor sequences will allow the expression of these receptors and the subsequent screening of agonists and antagonists. The identification of taxa- or species-specific ligands will aid in the development of more specific and safer acaricides. Combined with the opportunity to gain an understanding of acaricide resistance mechanisms, the development of new acaricides is important for the continued control of the cattle tick, *Rhipicephalus (Boophilus) microplus*, responsible for an estimated US$ 2 billion in annual economic losses worldwide [30].

## Materials and Methods

### cDNA Synthesis

Total RNA was purified from unfed tick larvae (Non Resistant Field Strain (NRFS)) [31] maintained at Biosecurity Science Laboratories (BSL) of the Department of Employment, Economic Development and Innovation (DEEDI) in Brisbane, Queensland. Approximately one gram of tick larvae was crushed under liquid nitrogen using a mortar and pestle. RNA extraction was performed using the TRIzol® Reagent (Invitrogen Life Technologies, Carlsbad, USA) following the manufacturer's protocol. Poly-A RNA was purified from total RNA using a POLY(A)Purist™ kit (Applied Biosystems/Ambion, Austin, USA). First strand cDNA synthesis was carried out using a Clontech SMARTer™ RACE cDNA Amplification Kit (Clontech Laboratories Inc. Mountain View, USA) following the manufacturer's directions.

### PCR and sequencing with degenerate primers

Amino acid sequences (Table S1) were aligned for each receptor of interest using ClustalW2 software (European Bioinformatics Institute, Cambridge, UK). Degenerate primers were designed (Figure 1) using CODEHOP software [32] from the aligned amino acid sequences. Each CODEHOP degenerate primer consisted of a pool of related primers containing all possible nucleotide sequences encoding 3–4 highly conserved amino acids within a 3′ degenerate tail. A longer 5′ non-degenerate clamp region contained the most probable nucleotides predicted for each flanking codon. The most probable nucleotides in the 5′ clamp for *Rhipicephalus microplus* were predicted from codon usage tables in CODEHOP. Wherever possible, primers chosen were specific to the receptor of interest only. Primer specificity was determined using the 3′ degenerate tail. (Figures 2 & 3) To determine the suitability for use with different templates, degenerate PCR was carried out on both gDNA and cDNA. If gDNA is the primary source of template, primers should be designed to amplify small fragments of less than 200 bp to increase the chance of both priming sites being contained within the same exon. 30 ng of template was amplified in a 25 µl reaction volume containing; 1 µl degenerate primers (primers were prepared at a concentration equal to 10 µM×level of degeneracy), 2 µl cDNA, 1× KAPA2G PCR Buffer B, 1× KAPA PCR Enhancer 1, 1.5 mM MgCl_2_, 0.2 mM dNTP and 0.05 U/µl KAPA2G Robust DNA Polymerase (KAPABiosystems. Woburn, USA). PCR conditions were: initial denaturation 95°C for 5 min, followed by 8 cycles of 94°C for 15 s, 63°C for 30 s decreasing by 1°C per cycle, 72°C for 2 min, followed by 27 cycles of 94°C for 15 s, 56°C for 30 s, 72°C for 2 min, followed by a final extension of 72°C for 7 min. PCR products were separated on a 1.5% agarose gel (Figure 4). Products of the correct size were sampled with a 10 µl pipette tip. This was used as template and re-amplified as above except with degenerate primers at a concentration of 10 µM. Unused dNTPs and primers were removed from the PCR product using Exosap-it® (USB Corporation distributed by GE Healthcare Bio-Sciences, Rydalmere, Australia). Sequencing was performed using an ABI Prism Big Dye Terminator Cycle Sequencing Ready Reaction Kit Version 3.1 (PE Applied Biosystems, Foster City, USA). The sequencing reaction contained 30 ng template DNA, 1 µl degenerate PCR primer (5 µM), 4.5 µl of 5× sequencing buffer, 1 µl Big Dye Terminator, to a final volume of 20 µl with MilliQ H_2_O. In a single case an internal degenerate sequencing primer was designed as both of the degenerate PCR primers failed to give clean sequence. This was used at a concentration equal to 5 µM×level of degeneracy. Sequencing separation was performed on an ABI 3130xl automated sequencer. Forward and reverse sequences were aligned and edited using ChromasPro (Technelysium Pty Ltd, Tewantin, Australia).

### RACE PCR

5RACE and 3RACE ready cDNA was prepared using a Clontech SMARTer™ RACE cDNA Amplification Kit following the manufacturer's directions. Using the sequences generated by degenerate PCR in the previous section and following the manufacturer's instructions, 3′ and 5′ gene specific RACE primers were designed and RACE PCR was performed. PCR products were separated on a 1.5% agarose gel. Products of the correct size were sampled with a 10 µl pipette tip and re-amplified. PCR products were cleaned and sequenced as described above. Internal sequencing primers were designed, where necessary, to enable sequencing of full length cDNAs.

## Supporting Information

Figures S1 **2 Dimensional representations of the eight isolated receptors, illustrating the 7 trans-membrane domains typical of all GPCR.** **A**) Rm_α2AOR: JN974908, **B**) Rm_β2AOR: JN974909, **C**) Rm_5HT_7_R: JN974910, **D**) Rm_INDR: JN974911, **E**) Rm_Dop1R: JN974914, **F**) Rm_Dop2R: JN974912 , **G**) Rm_mAchR: JN974913, **H**) Rm_GABA_B_R: JN974907. Membrane spanning domains were predicted by the TMHMM Server at the Center for Biological Sequence Analysis, Technical University of Denmark, DTU (http://www.cbs.dtu.dk/services/TMHMM/). 2 dimensional representation by TMRPres2D [34].
(TIF)

Figure S2 **Alignment of Class A GPCR, indicating features conserved in Class A GPCR.** Hs_mAchR_*Homo sapiens*_ Muscarinic acetylcholine receptor: ACE86798. Rm_mAchR_*Rhipicephalus* (*Boophilus) microplus_*Muscarinic acetylcholine receptor: JN974913. Membrane spanning residues are marked TM followed by the corresponding Roman numeral. Residues involved in ligand binding are highlighted in grey. Cysteines involved in forming a disulphide bond are in white text highlighted in black. Residues involved in receptor activation are in bold italics and underlined.
(DOC)

Figure S3 **Alignment of biogenic amine receptors. Indicating features conserved in biogenic amine receptors.** Hs_β2AR- *Homo sapiens_* β2-adrenergic Receptor: AAN01267. Rm_β2AOR_ *Rhipicephalus* (*Boophilus) microplus* β2-adrenergic-like octopamine receptor: JN974909. Membrane spanning residues are marked TM followed by the corresponding Roman numeral. Residues involved in ligand binding are highlighted in grey. Cysteines involved in forming a disulphide bond are in white text highlighted in black. Residues involved in receptor activation are in bold italics and underlined.
(DOC)

Figure S4 **Features conserved across biogenic amine GPCR.** Residues involved in ligand binding are highlighted in grey. Cysteines involved in forming a disulphide bond are in white text highlighted in black. Residues involved in receptor activation are in bold italics and underlined.
(DOC)

Figure S5 **Alignment of GABA_B_- receptors indicating features conserved in class C GPCR.** Hs_GABA_B_R _*Homo sapiens*_GABA_B_ receptor: CAA09940, Rm_GABA_B_R _*Rhipicephalus (Boophilus) microplus*_GABA_B_ receptor: JN974907 . Signal peptide sequences were predicted using SignalP 3.0. [35] Coiled-coil domains were predicted using COILS [36].
(DOC)

Table S1 **Accession numbers for amino acid and nucleic acid sequences used for designing degenerate primers.**
(DOC)

Table S2 **Accession numbers of sequences used for constructing the radial phylogram (** **Figure 5** **).**
(DOC)

## References

[1] Bockaert J, Pin JP (1999). Molecular tinkering of G protein coupled receptors: an evolutionary success.. EMBO J.

[2] Gilman AG (1987). G proteins: transducers of receptor-generated signals.. Annu Rev Biochem.

[3] Robas N, O'Reilly M, Katugampola S, Fidock M (2003). Maximizing serendipity: strategies for identifying ligands for orphan G-protein-coupled receptors.. Curr Opin Pharmacol.

[4] Flower DR (1999). Modelling G-protein-coupled receptors for drug design.. Biochim Biophys Acta.

[5] Baxter GD, Barker SC (1999). Isolation of a cDNA from an octopamine-like, G protein-coupled receptor from the cattle fever tick, Boophilus microplus.. Insect Biochem Molec Biol.

[6] Chen A, Holmes SP, Pietrantonio PV (2004). Molecular cloning and functional expression of a serotonin receptor from the southern cattle tick, Boophilus microplus (Acari: Ixodidae).. Insect Mol Biol.

[7] Larhammar D (1996). Structural diversity of receptors for neuropeptide Y, peptide YY and pancreatic polypeptide.. Reg Pept.

[8] Gether U (2000). Uncovering molecular mechanisms involved in activation of G protein-coupled receptors.. Endocrine Rev.

[9] Gether U, Kobilka BK (1998). G protein-coupled receptors. II. Mechanism of agonist activation.. J Biol Chem.

[10] Dixon RA, Sigal I, Candelore MR, Register RB, Scattergood W (1987). Structural features required for ligand binding to the beta-adrenergic receptor.. EMBO J.

[11] Schöneberg T, Schultz G, Gudermann T (1999). Structural basis of G protein-coupled receptor function.. Mol Cell Endocrinology.

[12] Strader CD, Fong TM, Graziano MP, Tota MR (1995). The family of G-protein-coupled receptors.. FASEB J.

[13] Evans PD (1980). Action of formamidine pesticides on octopamine receptors,. Nature.

[14] Hollingworth RM, Lund AE (1982). Biological and neurotoxic effect of amidine pesticides.. Insecticide Mode of Action.

[15] Roeder T (2005). Tyramine and octopamine: ruling behavior and metabolism. Annu.. Rev Entomol.

[16] Han KA, Millar NS, Davis RL (1998). A novel octopamine receptor with preferential expression in Drosophila mushroom bodies.. J Neurosci.

[17] Evans PD, Maqueira B (2005). Insect octopamine receptors: A new classification scheme based on studies of cloned Drosophila G-protein coupled receptors.. Invert Neurosci.

[18] Claassen DE, Kammer AE (1985). Effects of octopamine, dopamine and serotonin on production of flight motor output by thoracic ganglia of Manduca sexta.. J Neurobiol.

[19] Mercer AR, Menzel R (1982). The effect of biogenic amines on conditioned and unconditioned responses to olfactory stimuli in the honeybee Apis mellifera.. J Comp Physiol A.

[20] Mustard JA, Beggs KT, Mercer AR (2005). Molecular biology of the invertebrate dopamine receptors.. Arch Insect Biochem Physiol.

[21] Menzel R, Michlesen B, Rueffer P, Sugawa M, Hertting G, Spatz HC (1988). Neuropharmacology of learning and memory in honey bees.. Modulation of Synaptic Transmission and Plasticity in Nervous Systems.

[22] Bigg DCH, Purvis SR (1976). Muscarinic agonists provide a new class of acaricide.. Nature.

[23] Dick MR, Dripps JE, Orr N (1997). Muscarinic agonists as insecticides and acaricides.. Pestic Sci.

[24] Heinrich R, Wenzel B, Elsner N (2001). A role for muscarinic excitation: Control of specific singing behavior by activation of the adenylate cyclase pathway in the brain of grasshoppers.. PNAS.

[25] O'Hara PJ, Sheppard PO, Thogersen H, Venezia D, Haldeman BA (1993). The ligand-binding domain in metabotropic glutamate receptors is related to bacterial periplasmic binding proteins.. Neuron.

[26] Kammerer RA, Frank S, Schulthess T, Landwehr R, Lustig A (1999). Heterodimerization of a functional GABA_B_ receptor is mediated by parallel coiled-coil α-helices.. Biochemistry.

[27] Kuner R, Koehr G, Gruenewald S, Eisenhardt G, Bach A (1999). Role of heterodimer formation in GABA_B_ receptor function.. Science.

[28] Mezler M, Mueller T, Raming K (2001). Cloning and functional expression of GABA_B_ receptors from Drosophila.. Eur J of Neurosci.

[29] Hosie AM, Aronstein K, Sattelle DB, Ffrench-Constant RH (1997). Molecular biology of insect neuronal GABA receptors.. Trends Neurosci.

[30] Grisi L (2002). Impacto econômico das principais ectoparasitoses em bovinos no Brasil.. A Hora Veterinária.

[31] Stewart N, Callow L, Duncalfe F (1982). Biological comparisons between a laboratory-maintained and recently generated field strain of Boophilus microplus.. The Journal for Parasitology.

[32] Rose TM, Schultz ER, Henikoff JG, Pietrokovski S, McCallum CM (1998). Consensus-degenerate hybrid oligonucleotide primers for amplification of distantly-related sequences.. Nucl Acids Res.

[33] Huson HH, Richter DC, Rausch C, Dezulian T, Franz M (2007). Dendroscope: An interactive viewer for large phylogenetic trees,. RMC Bioinformatics.

[34] Spyropoulos IC, Liakopoulos TD, Pantelis GB, Hamodrakas SJ (2004). TMRPres2D: high quality visual representation of transmembrane protein models.. Bioinformatics.

[35] Bendtsen JD, Nielsen H, von Heijne G, Brunak S (2004). Improved prediction of signal peptides: SignalP 3.0.. J Mol Biol.

[36] Lupas A, Van Dyke M, Stock J (1991). Predicting Coiled Coils from Protein Sequences,. Science.

