# Supplementary figures and images for: Generation of Full-Length cDNAs for Eight Putative GPCnR from the Cattle Tick, R. microplus Using a Targeted Degenerate PCR and Sequencing Strategy
Source: PLoS One. 2012 Mar 5;7(3):e32480. doi: 10.1371/journal.pone.0032480 (PMC3293813; doi:10.1371/journal.pone.0032480)

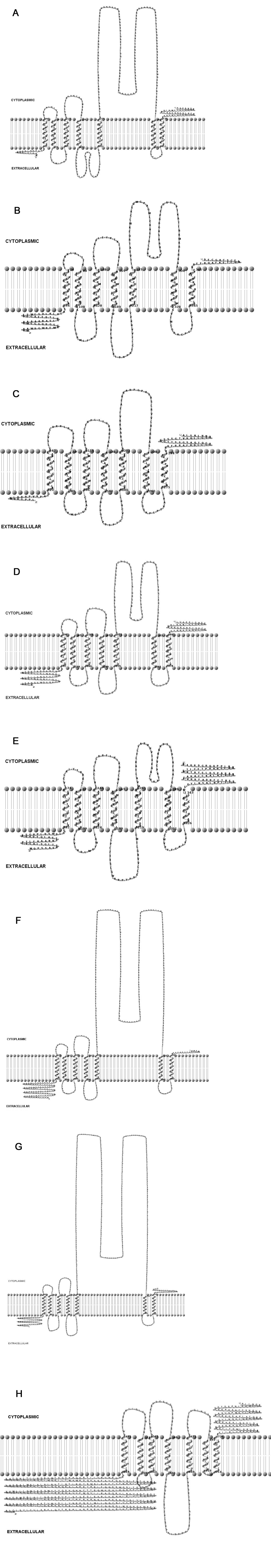

Supplement: Figures S1 — 2 Dimensional representations of the eight isolated receptors, illustrating the 7 trans-membrane domains typical of all GPCR. A) Rm_α2AOR: JN974908, B) Rm_β2AOR: JN974909, C) Rm_5HT7R: JN974910, D) Rm_INDR: JN974911, E) Rm_Dop1R: JN974914, F) Rm_Dop2R: JN974912 , G) Rm_mAchR: JN974913, H) Rm_GABABR: JN974907. Membrane spanning domains were predicted by the TMHMM Server at the Center for Biological Sequence Analysis, Technical University of Denmark, DTU (http://www.cbs.dtu.dk/services/TMHMM/). 2 dimensional representation by TMRPres2D [34]. (TIF) [file pone.0032480.s001.tif]
