# Supplementary material for: Generation of Full-Length cDNAs for Eight Putative GPCnR from the Cattle Tick, R. microplus Using a Targeted Degenerate PCR and Sequencing Strategy
Source: PLoS One. 2012 Mar 5;7(3):e32480. doi: 10.1371/journal.pone.0032480 (PMC3293813; doi:10.1371/journal.pone.0032480)
Supplement: Figure S2 — Alignment of Class A GPCR, indicating features conserved in Class A GPCR. Hs_mAchR_Homo sapiens_ Muscarinic acetylcholine receptor: ACE86798. Rm_mAchR_Rhipicephalus (Boophilus) microplus_Muscarinic acetylcholine receptor: JN974913. Membrane spanning residues are marked TM followed by the corresponding Roman numeral. Residues involved in ligand binding are highlighted in grey. Cysteines involved in forming a disulphide bond are in white text highlighted in black. Residues involved in receptor activation are in bold italics and underlined. (DOC) [file pone.0032480.s002.doc]

Hs_mAchR MTLHNNSTTSPLFPNISSSWIHSPSDAGLPPGTVTHFGSYNVSRAAGNFSSPDGTTDDPL 60

Rm_mAchR MGLLETALNASVLLAATEAGGSGGWNGVLDDGSGGASTTSGTGNAT-NDSVASGSSGHSA 59

* * :.: .:.:: :.: . :. * *: : ....*: * * ..*::...

________ TM I__________ ________TM II___

Hs_mAchR GGHTVWQVVFIAFLTGILALVTIIGNILVIVSFKVNKQLKTVNNYFLLSLACADLIIGVI 120

Rm_mAchR P-YSLPEVILIAFLAALLSAVTIIGNLMVMISFKLDKQLQTISNYFLLSLAIADFSIGVI 118

::: :*::****:.:*: ******::*::***::***:*:.******** **: ****

___________ _______**TM III**______

Hs_mAchR SMNLFTTYIIMNRWALGNLACDLWLAIDYVASNASVMNLLVISF***DRY***FSITRPLTYRAKR 180

Rm_mAchR SMPLFTMYTLYDHWPLGPFICDTWLAFDYLTSNASVLNLLIISF***DRY***FSVTRPLTYRARR 178

** *** * : ::*.** : ** ***:**::*****:***:********:********:*

_______ **TM IV**______ _________

Hs_mAchR TTKRAGVMIGLAWVISFVLWAPAILFWQYFVGKRTVPPGECFIQFLS-EPTITFGTAIAA 239

Rm_mAchR TTKRAAIMIASAWVISLVLWPPWIYSWPYIEGQRSVPLDRCYIQFLETNIYVTFGTALAA 238

*****.:**. *****:***.* * * *: *:*:** ..*:****. : :*****:**

___**TM V**_____

Hs_mAchR FYMPVTIMTILYWRIYKETEKRTKELAGLQASGTEAETENFVHPTGSSRSCSSYELQQ-- 297

Rm_mAchR FYVPVTVMCILYWRIWRETEKRQKDLTQLQAG--RKETGGSRKSTSSDDPAESEDFRRGR 296

**:***:* ******::***** *:*: ***. . ** . :.*.*. ...* ::::

Hs_mAchR -QSMKRSNRRKYGRCHFWFTTKSWKPSSE--------------QMDQDHSSSDSWNNNDA 342

Rm_mAchR SDSCPPDVETTYVPTSLCVETSKYLPPAAPKRRRLRDVLLSWCRIDNDKEDDDSTSHGGS 356

:* . . .* : . *..: *.: ::*:*:...** .:..:

Hs_mAchR AASLENSASSDEEDIGSET-RAIYSIVLKLPGHSTILNSTKLPSSDN--------LQVPE 393

Rm_mAchR PGTQTPASIETPVQSASMTFRADQLVQLNPAGRTGTGTSSAIASIGGGGGGGTGGVSIPM 416

..: :: . : .* * ** : *: .*:: .*: :.* .. :.:*

Hs_mAchR EELGMVDLERKADKLQAQKSVD---DGGSFPKSFSKLPIQ---------------LESAV 435

Rm_mAchR TDRNGLRRNEKTGATAAATSASRSYSSDSVYTILIRLPTQPSLEGETSQASIKMILEEDA 476

: . : :.*:. * .*.. ...*. . : :** * **. .

Hs_mAchR DT----------------AKTSDVNSSVGKST-----ATLPLSFKEATLAKRFALKTRSQ 474

Rm_mAchR EKHEAAGGAASSGAAATFARTSSEDSTVAMRTGDSGLETIRIPLNTKLVHRQVVAKQRAA 536

:. *:**. :*:*. * *: :.:: : ::.. * *:

**_________TM VI__________ TM VII_**

Hs_mAchR ITK-RKRMSLVKEKKAAQTLSAILLAFIITWTPYNIMVLVNTF--CDSCIPKTFWNLGYW 531

Rm_mAchR PKKKRKQQERKQEKKAAKTLSAILLAFIVTWTPYNVLVLIKTVSSCDDCIPTGLWNFVYY 596

.* **: . :*****:**********:******::**::*. **.***. :**: *:

________

Hs_mAchR LCYINSTVNPVCYALCNKTFRTTFKMLLLCQCDKKKRRKQQYQQRQSVIFHKRAPEQA 589

Rm_mAchR LCYINSTVNPLCYALCNANFRRTYMRILSCKWHNKQRSMNRG------YFT------- 641

**********:****** .** *: :* *: .:*:* :: *
