# Supplementary material for: Generation of Full-Length cDNAs for Eight Putative GPCnR from the Cattle Tick, R. microplus Using a Targeted Degenerate PCR and Sequencing Strategy
Source: PLoS One. 2012 Mar 5;7(3):e32480. doi: 10.1371/journal.pone.0032480 (PMC3293813; doi:10.1371/journal.pone.0032480)
Supplement: Figure S3 — Alignment of biogenic amine receptors. Indicating features conserved in biogenic amine receptors. Hs_β2AR- Homo sapiens_ β2-adrenergic Receptor: AAN01267. Rm_β2AOR_ Rhipicephalus (Boophilus) microplus β2-adrenergic-like octopamine receptor: JN974909. Membrane spanning residues are marked TM followed by the corresponding Roman numeral. Residues involved in ligand binding are highlighted in grey. Cysteines involved in forming a disulphide bond are in white text highlighted in black. Residues involved in receptor activation are in bold italics and underlined. (DOC) [file pone.0032480.s003.doc]

**________TM I________**

Hs_β2AR MGQPGNGSAFLLAPNRSHAPDHDVTQQRDEVWVVGMGIVMSLIVLAIVFGNVLVITAIAK 60

Rm_β2AOR MSAAAAANASLGNLTDEEAGDDESWESVLKLVLKTLALVT--IISSAVFGNLLVVTSVMR 58

*. .. ..* * . ..* *.: :. :: : :.:* *: : ****:**:*:: :

**___________TM II__________**  **TM III___**

Hs_β2AR FERLQTVTNYFITSLACA***D***LVMGLAVVPFGAAHILMKMWTFGNFWCEFWTSIDVLCVTAS 120

Rm_β2AOR HHKLRITTNYFIVSLALA***D***TLVALFAMTFNASVTISGRWLFNQTVCDFWNSCDVLFSTAS 118

..:*: .*****.*** ** ::.* .:.*.*: : * *.: *:**.* *** ***

_________ ___________**TM IV**__________

Hs_β2AR IETLCVIAV***DRY***FAITSPFKYQSLLTKNKARVIILMVWIVSGLTSFLPIQMHWYRATHQE 180

Rm_B2AOR IMHLCCISV***DRY***YAIIKPLEYPTKITGRTVAIMLACAWISSGLMSFMPIFMGWY-TTDEH 177

* ** *:****:** .*::* : :* ... ::: .** *** **:** * ** :*.:.

**________TM V_______**

Hs_β2AR AINCYANETCCDFFTNQAYAIASSIVSFYVPLVIMVFVYSRVFQEAKRQLQKIDKSEG-- 238

Rm_β2AOR LGYKLDHPDECMFVVNKPYAMVSSSVSFWIPCCIMLFTYWRIYVEATRQEKMLCKSQMGP 237

: * *..*:.**:.** ***::* **:*.* *:: **.** : : **:

**__TM VI__**

Hs_β2AR -----RFHVQNLSQVEQDGRTGHG----------LRRSSKFCLKEHKALKTLGIIMGTFT 283

Rm_β2AOR AGMLCRNSTDLPNVHAPPHRNSHGDDPESGQSTPTKRTINKMKREHKAAKTLGIIMGAFI 297

* .: . *..** :*: : :**** ********:*

___________ **______TM VII______**

Hs_β2AR LCWLPFFIVNIVHVIQDN--LIRKEVYILLNWIGYVNSGFNPLIYCRS-PDFRIAFQELL 340

Rm_β2AOR LCWLPFFLWYVSVTMCGDACPCPDLVVDLLFWIGYLNSSLNPVIYAYFNSEFRQAFKETL 357

*******: : .: .: . * ** ****:**.:**:**. .:** **:* *

Hs_β2AR CLRRSSLKAYGNGYSSNGNTGEQSGYHVEQEKENKLLCEDLPGTEDFVGHQGTVPSDNID 400

Rm_β2AOR QAIFCSCAGCEDWVGSQ---------RWLASSWHRYSCEDTG--PGFVEQHRFVDTRPAA 406

.* . : .*: : .. :: *** .** :: * :

Hs_β2AR SQGRNCSTNDSLL 413

Rm_β2AOR AQSDWTALA---- 415

:*. :
