# Supplementary material for: Generation of Full-Length cDNAs for Eight Putative GPCnR from the Cattle Tick, R. microplus Using a Targeted Degenerate PCR and Sequencing Strategy
Source: PLoS One. 2012 Mar 5;7(3):e32480. doi: 10.1371/journal.pone.0032480 (PMC3293813; doi:10.1371/journal.pone.0032480)
Supplement: Figure S4 — Features conserved across biogenic amine GPCR. Residues involved in ligand binding are highlighted in grey. Cysteines involved in forming a disulphide bond are in white text highlighted in black. Residues involved in receptor activation are in bold italics and underlined. (DOC) [file pone.0032480.s004.doc]

**____________TM_II_________ _______TM III______**

Rm_α2AOR YFVVSLAVA***D***LSVGLTVLPYSIVLEVL-EVWIFGHTWCQIWLAVDVWLCTSSILNLCAISV***DRY***LAIT

Rm_INDR YFIASLATA***D***CLVGAVVMPFSAIHEIMDKYWIFGQDLCDVWHSIDVLASTASILNLCVISL***DRY***WAIT

Rm_β2AOR YFIVSLALA***D***TLVALFAMTFNASVTISGR-WLFNQTVCDFWNSCDVLFSTASIMHLCCISV***DRY***YAII

Rm_Dop1R LFLVSLAVA***D***LLVSSLVMTFAVINDLMGY-WAFGPQFCDIWIAFDVMCSTASILNLCAISL***DRF***LHIK

Rm_Dop2R YFIVSLAFA***D***LLVAAAVMPFAVYVLVNVD-WELSETLCDFYIAVDVTCSTASIFNLVAISI***DRF***IAVT

Rm_5-HT_7_R -LLVSLAAS***D***LCVALLVMPPAMYLELSGHRWDLGRAACDAWVSMDVASCTASILNLCMISV***DRY***LAIT

::**** :* :. :. : * :. *: * : ** .*:**::* **.**

________**TM V**_______ _________**TM VI**______

Rm_α2AOR SAQCVLINNKGYVIYSALGSFYIPMLFMLFFNYRIYRAA KATKTVGTIVGGFICCWLPFFTVYLVRAF

Rm_INDR AFQCAFTDDVGYLVFSSTISFYAPLMVMVFTYYRIYRAA KAAKTLAIVMGVFILCWLPFFVTNILMGI

Rm_β2AOR PDECMFVVNKPYAMVSSSVSFWIPCCIMLFTYWRIYVEA KAAKTLGIIMGAFILCWLPFFLWYVSVTM

Rm_Dop1R LTMCALDLTPEYAVTSSLISFYMPCVVMVALYARLYLYA KAAITLGIIVGVFLCCWVPFFCANIVAAF

Rm_Dop2R PHLCIFY-NSDFILYSSLSSFYIPCLVMVFLYYKIFRVM KATKTLAIVLGVFLICWVPFFTCNVVDAV

Rm_5-HT_7_R SPTCLVCQHLAYQLY**A**TLG**A**FYMPLAVMLFVYWSMHSAA KASITLGIILTAFTACWLPFFALALVRPL

Rm_5-HT_1_R EKKCLVSQDAAYQVF**A**TCSSFYVPLIMILLLYWRIFKVA **: *:. :: * ** *** : :

* . : : :: :*: * .:: :.
