# Supplementary material for: Generation of Full-Length cDNAs for Eight Putative GPCnR from the Cattle Tick, R. microplus Using a Targeted Degenerate PCR and Sequencing Strategy
Source: PLoS One. 2012 Mar 5;7(3):e32480. doi: 10.1371/journal.pone.0032480 (PMC3293813; doi:10.1371/journal.pone.0032480)
Supplement: Figure S5 — Alignment of GABAB- receptors indicating features conserved in class C GPCR. Hs_GABABR _Homo sapiens_GABAB receptor: CAA09940, Rm_GABABR _Rhipicephalus (Boophilus) microplus_GABAB receptor: JN974907 . Signal peptide sequences were predicted using SignalP 3.0. [35] Coiled-coil domains were predicted using COILS [36]. (DOC) [file pone.0032480.s005.doc]

Hs_GABA_B_R MGPGAPFARVGWPLPLLVVMAAGVAPVWASHSPHLPRPHSRVPPHPSSERRAVYIGALFP 60

Rm_GABA_B_R -------MDCAICLSVLCVLAR---FLQVTRSP-LANPNEKSRDKEYKTSKSLYIAAVFP 49

. *.:* *:* : .::** *..*:.: : . :::**.*:**

Signal Peptide

Hs_GABA_B_R MS--GGWPGGQACQPAVEMALEDVNSRRDILPDYELKLIHHDSKCDPGQATKYLYELLYN 118

Rm_GABA_B_R MKGHGGWLGGQGCFPAALMALEDVNKRSDLLIGYKLEIDWRDSQCNPGLAATVMYDLLYN 109

*. *** ***.* **. *******.* *:* .*:*:: :**:*:** *:. :*:****

Hs_GABA_B_R DPIKIILMPGCSSVSTLVAEAARMWNLIVLSYGSSSPALSNRQRFPTFFRTHPSATLHNP 178

Rm_GABA_B_R EPQKLMLLGGCSIVCSTVAEAAKMWNLVVISYGSSSPALSNRKRFPTFFRTHPSATIHNP 169

:* *::*: *** *.: *****:****:*:************:*************:***

Hs_GABA_B_R TRVKLFEKWGWKKIATIQQTTEVFTSTLDDLEERVKEAGIEITFRQSFFSDPAVPVKNLK 238

Rm_GABAbR TRIKLFQKFSWSRIAIIQEAEEVFISTGEDLEARCKEAHIEIVTRQSFLTDPTDAVKNLV 229

**:***:*:.*.:** **:: *** ** :*** * *** ***. ****::**: .****

Hs_GABA_B_R RQDARIIVGLFYETEARKVFCEVYKERLFGKKYVWFLIGWYADNWFKIYDPSINCTVDEM 298

Rm_GABA_B_R RQDARIIVGMFYVAAARRVFCEAYKQNVFGKQYVWLLIGWYEDGWYTVQDKGHNCTTEQM 289

*********:** : **:****.**:.:***:***:***** *.*:.: * . ***.::*

Hs_GABA_B_R TEAVEGHITTEIVMLNPANTRSISNMTSQEFVEKLTKRLKRHP-----EETGGFQEAPLA 353

Rm_GABA_B_R KEALEGHFTTEALMLNQGSQETISGMSSQQFLERYERALAEQNGGLQGYKPEGHQEAPLA 349

.**:***:*** :*** .. .:**.*:**:*:*: : * .: :. *.******

Hs_GABA_B_R YDAIWALALALNKTSGGGGRSGVRLEDFNYNNQTITDQIYRAMNSSSFEGVSGHVVFDAS 413

Rm_GABA_B_R YDAIWAIALALNKTINTLREYSMSIEDFTYTNHKIADEIWSAMNATQFLGVSGFVAFSAK 409

******:******* . . .: :***.*.*:.*:*:*: ***::.* ****.*.*.*.

___

Hs_GABA_B_R GSRMAWTLIEQLQGGSYKKIGYYDSTKDDLSWSKTDKWIGGSPPADQTLVIKTFRFLSQK 473

Rm_GABA_B_R GDRMAWTLIEQMIDGNYVKIGYFDTQTDNLTILNQEKWTDGKPPQDRTIIVRVHRKVSLS 469

*.*********: .*.* ****:*: .*:*: : :** .*.** *:*::::..* :* .

**_________TM I___________ ________TM II________**

Hs_GABA_B_R LFISVSVLSSLGIVLAVVCLSFNIYNSHVRYIQNSQPNLNNLTAVGCSLALAAVFPLGLD 533

Rm_GABA_B_R LFAGMCAVAFIGVVWAVGLLIFNWIFRHSRYIQLSHPMCNNIMLIGIILCLVCVCLLGLD 529

** .:..:: :*:* ** * ** * **** *:* **: :* *.*..* ****

____ _________**TM III**_______

Hs_GABA_B_R GYHIGRNQFPFVCQARLWLLGLGFSLGYGSMFTKIWWVHTVFTKK--EEKKEWRKTLEPW 591

Rm_GABA_B_R GQFVSEFRYAHICQARSWFLAIGFTLSFGAMFSKIWRVHRLTTKSKSESKGLSFQRVESW 589

* .:.. ::..:**** *:*.:**:*.:*:**:*** ** : **. *.* : :*.*

_________**TM IV**________ _

Hs_GABA_B_R KLYATVGLLVGMDVLTLAIWQIVDPLHRTIETFAKEEPKE-DIDVSILPQLEHCSSRKMN 650

Rm_GABA_B_R RLYGMVGGLVLVDAVILSAWQLVDPMQRHLEVFPLEPPALSDEDVRIEPALEHCESRNHA 649

:**. ** ** :*.: *: **:***::* :*.*. * * * ** * * ****.**:

_________**TM V**________ _________**TM VI**________

Hs_GABA_B_R TWLGIFYGYKGLLLLLGIFLAYETKSVSTEKINDHRAVGMAIYNVAVLCLITAPVTMILS 710

Rm_GABA_B_R IWLGVMYSYKGLLLIFGIFLAYETRSVKIKQLNDSRLVGMSIYNVVVLCLITAPVTLVIG 709

***::*.******::********:**. :::** * ***:****.**********:::.

_________**TM VII**___________

Hs_GABAbR SQQDAAFAFASLAIVFSSYITLVVLFVPKMRRLITRGEWQSEAQDTMKTGSSTNNNEEEK 770

Rm_GABAbR SQQDATFAFVALAIIFCSFLSMALIFVPKIIELVRRPRERADVRSLMDT--ITSKEEEER 767

*****:***.:***:*.*::::.::****: .*: * . :::.:. *.* *.::***:

Hs_GABA_B_R SRLLEKENRELEKIIAEKEERVSELRHQLQSRQQLRSRRHPPTP---------------- 814

Rm_GABA_B_R HQRLLAENEDLKKQIAEKEEQIQVLNQKLQERQRLAHQTALPASGERVFLASVSPWSVLA 827

: * **.:*:* ******::. *.::**.**:* : *:.

Coiled-coil-domain

Hs_GABA_B_R PEPSGGLPRGPPEPPDRLSCDGSRVHLLYK------- 844

Rm_GABA_B_R PDTLVGHSCCAAAPEEYTKTDSPNLRHMCIISGNIIP 864

*:. * . .. * : . *...:: :
