# Supplementary material for: Generation of Full-Length cDNAs for Eight Putative GPCnR from the Cattle Tick, R. microplus Using a Targeted Degenerate PCR and Sequencing Strategy
Source: PLoS One. 2012 Mar 5;7(3):e32480. doi: 10.1371/journal.pone.0032480 (PMC3293813; doi:10.1371/journal.pone.0032480)
Supplement: Table S1 — Accession numbers for amino acid and nucleic acid sequences used for designing degenerate primers. (DOC) [file pone.0032480.s006.doc]

**Table S1.**

Accession numbers for amino acid and nucleic acid sequences used for designing degenerate primers.

|  | **αAOR** | **βAOR** | **Dop1R** | **Dop2R** | **INDR** | **GABA_B_R** | **mAchR** | **5HT_7_R** |
| --- | --- | --- | --- | --- | --- | --- | --- | --- |
| **Ag** | XP_311113 |  | XP_315207 | NM_001014983 | XP_311193 | XM_319474 | XP_314486 | XP_313127 |
|  |  |  |  |  |  |  |  |  |
| **Am** | NP_001011565 | XP_396348 | NP_001011595 |  | NP_001011567 | XM_392294 | XP_395760 | NP_001071289 |
|  |  |  |  |  |  |  |  |  |
| **Ap** |  | XP_001944827 |  | XM_001952237 |  | XM_001952406 |  |  |
|  |  |  |  |  |  |  |  |  |
| **Dm** | NP_732541 | NP_001034049 | CAA54451 | AY150864 | NP_524548 | NM_078845 |  | NP_524599 |
|  |  |  |  |  |  |  |  |  |
| **Is** | XP_002408812 | XP_002411135 | XP_002409287 | XM_002416405 | XP_002399655 | XM_002406043 | XP_002403135 | XP_002435581 |
|  |  |  |  |  |  |  |  |  |
| **Nv** |  | XP_001606684 | XP_001606438 | XM_001602460 | NP_001155849 | XM_001605233 |  | XP_001606275 |
|  |  |  |  |  |  |  |  |  |
| **Tc** | [XP_970007](http://www.ncbi.nlm.nih.gov/entrez/query.fcgi?cmd=Retrieve&db=Protein&list_uids=189241401&dopt=GenPept&RID=1KGW38DG014&log$=protalign&blast_rank=2) | XP_974214 | XP_971542 | XM_963944 | XP_972779 |  | XP_972657 | XP_966577 |

Ag-*Anopheles gambiae*, Am-*Apis mellifera*, Ap-*Acyrthosiphon pisum*, Dm-*Drosophila melanogaster*, Is-*Ixodes scapularis*, Nv-*Nasonia vitripennis*, Tc-*Tribolium castaneum*
