# Supplementary material for: Generation of Full-Length cDNAs for Eight Putative GPCnR from the Cattle Tick, R. microplus Using a Targeted Degenerate PCR and Sequencing Strategy
Source: PLoS One. 2012 Mar 5;7(3):e32480. doi: 10.1371/journal.pone.0032480 (PMC3293813; doi:10.1371/journal.pone.0032480)
Supplement: Table S2 — Accession numbers of sequences used for constructing the radial phylogram ( Figure 5 ). (DOC) [file pone.0032480.s007.doc]

**Table S2**. Accession numbers of sequences used for constructing the radial phylogram (Figure 5)

|  | **αAOR** | **βAOR** | **Dop1R** | **Dop2R** | **INDR** | **GABA_B_** | **mAchR** | **5HT_7_R** | **5HT_1_R** | **Oct/TyrR** |
| --- | --- | --- | --- | --- | --- | --- | --- | --- | --- | --- |
| **Ag** | XP_311113 |  | XP_315207 |  | XP_311193 | XM_319474 | XP_314486 | XP_313127 | XP_308623 | XP_312420 |
|  |  |  |  |  |  |  |  | XP_313129 |  |  |
|  |  |  |  |  |  |  |  |  |  |  |
| **Am** | NP_001011565 | XP_396348 | NP_001011595 | NP_001014983 | NP_001011567 | XM_392294 | XP_395760 |  | XP_393915 | NP_001011594 |
|  |  | XP_397139 |  |  |  |  |  | NP_001071289 |  |  |
|  |  | XP_397077 |  |  |  |  |  |  |  |  |
|  |  |  |  |  |  |  |  |  |  |  |
| **Ap** |  | XP_001944827 |  | XP_001952272 |  | XM_001952406 |  | XP_001945446 |  |  |
|  |  | XP_001947781 |  |  |  |  |  |  |  |  |
|  |  |  |  |  |  |  |  |  |  |  |
| **Rm** | JN974908 | JN974909 | JN974914 | JN974912 | JN974911 | JN974907 | JN974913 | JN974910 | AAQ89933 | CAA09335 |
|  |  |  |  |  |  |  |  |  |  |  |
| **Dm** | NP_732541 | NP_001034049 | CAA54451 |  | NP_524548 | NM_078845 |  | NP_524599 | NP_001137708 | NP_524419 |
|  |  | NP_001034043 |  | [NP_001014758](http://www.ncbi.nlm.nih.gov/entrez/query.fcgi?cmd=Retrieve&db=Protein&list_uids=62473889&dopt=GenPept&RID=CSJ3HJ4K012&log$=protalign&blast_rank=3) |  |  |  |  |  |  |
|  |  | NP_651057 |  |  |  |  |  |  |  |  |
|  |  |  |  |  |  |  |  |  |  |  |
| **Is** | XP_002408812 | XP_002411135 | XP_002409287 | [XP_002416450](http://www.ncbi.nlm.nih.gov/entrez/query.fcgi?cmd=Retrieve&db=Protein&list_uids=241811617&dopt=GenPept&RID=CNS514CA01N&log$=prottop&blast_rank=1) | XP_002399655 | XM_002406043 | XP_002403135 | XP_002435581 | XP_002405023 | XP_002408737 |
|  |  |  |  |  |  |  |  | XP_002435580 | XP_002404998 | XP_002415939 |
|  |  |  |  |  |  |  |  |  |  |  |
| **Nv** |  | XP_001606684 | XP_001606438 | XP_001602510 | NP_001155849 | XM_001605233 |  | XP_001606275 | XP_001603891 |  |
|  |  |  |  |  |  |  |  |  |  |  |
| **Pa** | AAP93817 |  |  |  |  |  |  |  | CAX65666 | CAQ48240 |
|  |  |  |  |  |  |  |  |  |  |  |
| **Pc** |  |  |  |  |  |  | ACE60222 |  |  |  |
|  |  |  |  |  |  |  |  |  |  |  |
| **Phc** |  |  |  |  |  |  | XP_002430487 |  |  |  |
|  |  |  |  |  |  |  |  |  |  |  |
| **Tc** | [XP_970007](http://www.ncbi.nlm.nih.gov/entrez/query.fcgi?cmd=Retrieve&db=Protein&list_uids=189241401&dopt=GenPept&RID=1KGW38DG014&log$=protalign&blast_rank=2) | XP_974214 | XP_971542 | XP_969037 | XP_972779 |  | XP_972657 | XP_966577 | XP_967449 | XP_970290 |
|  |  | XP_974265 |  |  |  |  |  |  | XP_972856 |  |
|  |  | XP_974238 |  |  |  |  |  |  |  |  |

Ag-*Anopheles gambiae*, Am-*Apis mellifera*, Ap-*Acyrthosiphon pisum*, Rm-*Rhipicephalus* (*Boophilus) microplus,*

Dm-*Drosophila melanogaster*, Is-*Ixodes scapularis*, Nv-*Nasonia vitripennis*, Pa-*Periplaneta Americana*, Pc-*Polyrhachis vicina*,

Phc-*Pediculus humanus corporis*, Tc-*Tribolium castaneum*,
